# Differential recovery of ventricular repolarization and hemodynamics following bupivacaine and ropivacaine intoxication: the impact of lipophilicity on lipid emulsion efficacy

**DOI:** 10.3389/fphar.2026.1812943

**Published:** 2026-06-18

**Authors:** Lucía Rodríguez-Rodríguez, Julio Orallo-Martínez, Ignacio Fernández-López, Elena Vázquez, Sergio García-Ramos, Oscar Quintela, Begoña Bravo, Sara Alvarez-Zaballos, María José Anadón-Baselga, Matilde Zaballos

**Affiliations:** 1 Department of Anesthesiology, Hospital General Universitario Gregorio Marañón, Madrid, Spain; 2 Hospital General Universitario Gregorio Marañón, Madrid, Spain; 3 Department of Toxicology, Faculty of Medicine, Complutense University, Madrid, Spain. National Institute of Toxicology and Forensic Science, Madrid, Spain; 4 National Institute of Toxicology and Forensic Science, Madrid, Spain; 5 Department of Cardiology, Hospital General Universitario Gregorio Marañón, Madrid, Spain; 6 Head Department of Toxicology, Faculty of Medicine, Complutense University, Madrid, Spain; 7 Department of Toxicology, Faculty of Medicine, Complutense University, Madrid, Spain; 8 Department of Anesthesiology, Hospital General Universitario Gregorio Marañón, Instituto de Investigation Sanitaria Gregorio Marañón, Madrid, Spain

**Keywords:** bupivacaine, lipid-emulsion, ropivacaine, swine model, ventricular-repolarization

## Abstract

**Introduction:**

Local anesthetic systemic toxicity (LAST) causes significant ventricular repolarization disturbances. While intravenous lipid emulsion (ILE) is the standard treatment, the factors governing the simultaneous recovery of electrical and mechanical functions remain unclear. We evaluated ILE efficacy in reversing repolarization alterations and hemodynamic collapse induced by clinically equipotent doses of bupivacaine and ropivacaine.

**Methods:**

Twelve swine were randomized to receive a toxic bolus of bupivacaine (4 mg/kg) or ropivacaine (5 mg/kg). The primary outcome was the Area Under the Curve (AUC) of the corrected T-peak to T-end interval (Tp-TeC) during the recovery phase. Secondary outcomes included QTc and mean blood pressure (MBP) AUCs. Analysis was divided into a toxicity induction phase (0–3 min) and a recovery phase (3–25 min) to integrate the magnitude and duration of the alterations.

**Results:**

Both anesthetics significantly impaired repolarization, with no differences in total toxic load during the initial phase (*p* > 0.05). Following ILE, a distinct pharmacological divergence occurred. Electrical recovery was significantly more effective in the bupivacaine group, with lower recovery AUC values for the primary outcome (Tp-TeC, *p* = 0.002) and QTc (*p* = 0.004). Conversely, hemodynamic rescue was more robust following ropivacaine intoxication (higher MBP recovery AUC, *p* = 0.025). Recovery of QRS duration and sinus cycle length was similar between groups.

**Conclusion:**

Our findings demonstrate a distinct pharmacological divergence in lipid resuscitation therapy. Bupivacaine’s higher lipophilicity (Log D) enhances electrical recovery via a more efficient “lipid shuttle” effect on ion channels, while ropivacaine’s lower intrinsic cardiotoxicity allows for a more resilient mechanical recovery. These results suggest that electrical and mechanical rescue are governed by different rules dictated by the anesthetic’s physicochemical properties.

## Introduction

Local anesthetic systemic toxicity (LAST) is a life-threatening complication that can induce malignant ventricular arrhythmias (Butterworth, 2010; Macfarlane et al., 2021; Singaravelu Ramesh and Boretsky, 2021; Fettiplace M. et al., 2025). While ultrasound-guided techniques have reduced inadvertent intravascular injections, systemic toxicity from the absorption of large drug depots remains a significant risk in contemporary practice (Fettiplace MR. et al., 2025). These agents cause ventricular repolarization disturbances by inhibiting cardiac potassium currents, particularly the rapid delayed rectifier current (IKr) generated by human Ether-à-go-go-Related Gene (hERG) channels, increasing the heterogeneity of the electrical substrate (Clarkson and Hondeghem, 1985; Kasten, 1986).

The T-peak to T-end (Tp-Te) interval has emerged as a robust indicator for assessing the transmural dispersion of repolarization (TDR) and serves as a key predictor of reentrant arrhythmias (Antzelevitch, 2005; Castro-Torres et al., 2015). Despite the clinical importance of bupivacaine and ropivacaine, their impact on advanced repolarization parameters and their response to lipid resuscitation therapy remain less thoroughly characterized (De Diego et al., 2019).

Bupivacaine and ropivacaine exhibit distinct lipophilicity profiles. The distribution coefficient at physiological pH (Log D at pH 7.4) is more clinically relevant for predicting drug behavior, with values of 2.63 for bupivacaine and 2.12 for ropivacaine (Wiedmer and Sohn, 2024). Since its inclusion in international practice advisories (Neal et al., 2018), intravenous lipid emulsion (ILE) has become the standard treatment for LAST. Current evidence supports a multi-modal mechanism for ILE, involving a dynamic “lipid shuttle” effect for drug partitioning and direct cardiotonic and metabolic actions (Fettiplace and Weinberg, 2018; Lee and Sohn, 2023; Ok et al., 2018).

To investigate these interactions, we employed a porcine model. Although pigs are susceptible to complement activation-related pseudoallergy (CARPA) following lipid infusion (Bedocs et al., 2014; Szebeni et al., 2012), the model offers high translational validity for cardiac electrophysiology due to its anatomical and ion-channel similarities to humans (Arlock et al., 2017; Swindle et al., 2012). Previous work by our group has confirmed the stability of this model for assessing the studied parameters (Zaballos et al., 2019).

While ILE efficacy is multi-modal, lipophilicity remains a significant factor in drug redistribution. We hypothesized that ILE would facilitate a more pronounced reversal of ventricular repolarization disturbances in bupivacaine intoxication compared to ropivacaine, given its higher Log D. The primary outcome was the recovery phase Area Under the Curve (AUC) of the corrected T-peak to T-end interval (Tp-TeC). Additionally, we aimed to investigate the relationship between this electrical restoration and the recovery of mean blood pressure (MBP).

## Materials and methods

### Ethical approval and animals

The experimental protocol (No. PROEX-260/16) was authorized by the Institutional Animal Care and Use Committee of Gregorio Marañón University Hospital and the Madrid Regional Environmental Department. Procedures adhered to the guidelines of the Spanish Ministry of Agriculture. Twelve Sach miniature pigs (5 males and 7 females), weighing 35–55 kg, were used. Animals were subjected to a 12-h fast with unrestricted access to water.

### Experimental protocol and anesthesia

The study utilized a validated closed-thorax porcine model, established for the electrophysiological assessment of local anesthetic toxicity (De Diego et al., 2019; Zaballos et al., 2019). This model is particularly suitable for assessing ventricular repolarization, and our previous work demonstrated that, at the doses used, ILE does not inherently alter the studied electrophysiological parameters in this species (Zaballos et al., 2019).

Animals were premedicated with intramuscular ketamine (20 mg kg^-1^) (Boschert et al., 1996) and induced with intravenous sodium thiopental. General anesthesia was maintained with sevoflurane at 2.6% inspired concentration (Manohar and Parks, 1984). Ventilation was adjusted (6–8 mL kg^-1^ tidal volume; 12 breaths/min) to maintain normocapnia. Physiological stability was supported with normal saline (5 mL kg^-1^·h^-1^), and body temperature was maintained near 38 °C using a forced-air warming system. Monitoring included pulse oximetry, end-tidal sevoflurane and carbon dioxide (CO_2)_, and fraction of inspired oxygen (FiO_2_). Vascular access was established via femoral artery and vein cannulation for continuous invasive blood pressure recording and drug administration.

### Randomization and intervention

Subjects were randomized (block randomization) to receive either bupivacaine or ropivacaine. The allocation sequence was maintained in opaque envelopes and revealed to investigators immediately prior to administration. Subjects received an initial intravenous bolus of 0.5% bupivacaine (4 mg/kg) or 0.75% ropivacaine (5 mg/kg), followed by a continuous maintenance infusion of 100 μg/kg/min to induce distinct electrophysiological alterations while avoiding mortality.

**FIGURE 1:**
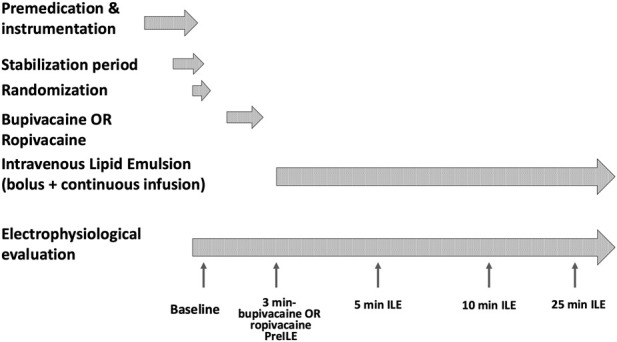
Schematic representation of the experimental protocol. The timeline illustrates the sequence of interventions and data acquisition points. Following instrumentation and a 10-min stabilization period, animals were randomized to receive a toxic intravenous bolus of either bupivacaine (4 mg/kg) or ropivacaine (5 mg/kg). Three minutes post-injection (Pre-ILE), rescue therapy with a 20% intravenous lipid emulsion (ILE) was initiated, consisting of a 1.5 mL/kg loading dose followed by a continuous infusion of 0.25 mL/kg/min. Vertical arrows indicate the predetermined intervals for electrophysiological and hemodynamic evaluations: baseline, peak intoxication (Pre-ILE), and 5, 10, and 25 min after the start of ILE administration.

Three minutes post-bolus, lipid resuscitation therapy was initiated with 20% ILE (Intralipid®). An initial loading dose of 1.5 mL/kg was administered over one minute, followed by a continuous infusion of 0.25 mL/kg/min maintained until the conclusion of the study (25 min post-ILE). This observation period was selected based on previous research showing that the most significant alterations typically resolve or reach a stable plateau within this timeframe (Zaballos et al., 2019). Upon completion of the experimental protocol, the animals were euthanized with a lethal dose of intravenous propofol followed by potassium chloride. The experimental sequence is summarized in Figure 1.

### Electrocardiographic and hemodynamic monitoring

A 12-lead ECG system was digitally recorded (LABSYSTEM PRO EP) at a sweep speed of 100 mm/s. All intervals were calculated as the mean of three consecutive cycles per lead using digital calipers. Parameters included.

**FIGURE 2:**
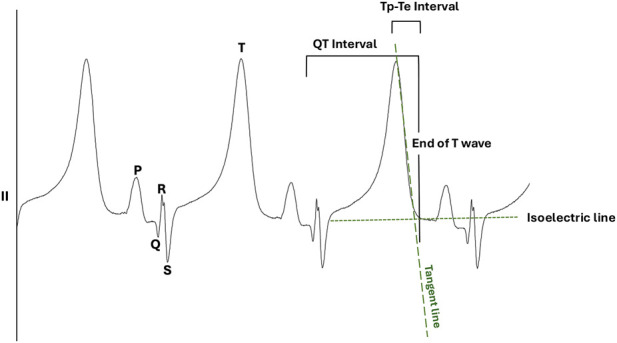
Schematic illustration of the electrocardiographic measurement methodology. The diagram demonstrates the identification of the P-QRS-T components and the calculation of the QT and T-peak to T-end (Tp-Te) intervals. The end of the T-wave is defined using the tangent method: the intersection between the isoelectric line and the tangent line drawn along the maximal downward slope of the T-wave. All measurements were performed at a sweep speed of 100 mm/s to ensure precision.

- Sinus cycle length (SCL): Measured as the RR interval.
- QRS duration: Measured from the earliest onset to the latest offset across all leads using superimposed complexes (Surawicz et al., 2009).
- QT and Corrected QT (QTc) intervals: QTc was calculated via Bazett’s formula (QT/√RR).
- T-peak to T-end (Tp-Te) and Corrected Tp-Te (Tp-TeC): Measured from peak to end of the T-wave (tangent method). Figure 2. Tp-TeC was calculated as ( $TpTe/\sqrt{RR}$ ).
- Dispersions (QTd, Tp-TeD): Difference between maximal and minimal intervals across the 12 leads.

To ensure stability, a minimum of five leads with discernible T-waves was required. ECG traces were analyzed independently by two blinded investigators. Inter-observer reliability, assessed via the intraclass correlation coefficient (ICC), was 0.91 at baseline and 0.96 during peak toxicity, indicating excellent agreement between investigators.

### Local anesthetic quantification

Arterial blood was sampled at baseline, 1, 5, 15, and 25 min post-bolus. Plasma concentrations were quantified using liquid chromatography-tandem mass spectrometry (LC-MS/MS) (Zaballos et al., 2016; Zaballos et al., 2019).

### Statistical analysis

Statistical analyses were performed using IBM SPSS Statistics 29.0. Data normality was assessed using the Shapiro–Wilk test. Continuous variables are presented as median and interquartile range (IQR). The experimental period was divided into two distinct phases for Area Under the Curve (AUC) analysis (trapezoidal rule): (1) the toxicity induction phase (from drug administration to the start of rescue, 0–3 min) and (2) the recovery phase (from the initiation of ILE therapy until the end of the study, 3–25 min).

The primary outcome was the recovery phase AUC of the Tp-TeC interval; secondary outcomes included QTc and MBP AUCs. Inter-group AUC comparisons were performed using the Mann-Whitney U test, and intra-group comparisons were conducted via the Wilcoxon signed-rank test. Inter-observer reliability for electrocardiographic measurements was evaluated using the intraclass correlation coefficient (ICC), employing a two-way mixed model with absolute agreement. Statistical significance was defined as a two-tailed p-value <0.05.

### Sample size calculation

The recovery phase AUC of the Tp-TeC was selected as the primary endpoint for the power analysis. Sample size was determined based on data obtained during an initial pilot phase of this experimental protocol, where a mean recovery phase AUC of 2,117 ± 251 ms⋅min was observed for bupivacaine and 2,952 ± 290 ms⋅min for ropivacaine. To detect a difference ≥500 ms⋅min in recovery AUC, with a common standard deviation of 290 ms⋅min, a power of 80%, and a significance level of 0.05, 6 subjects per group were required. A 10% dropout rate was anticipated, resulting in a total sample size of 12 animals.

## Results

### Baseline characteristics and laboratory parameters

**FIGURE 3:**
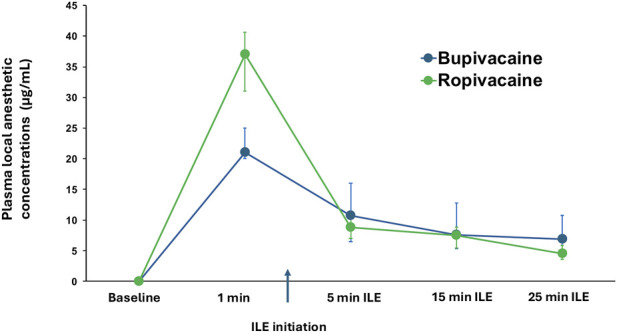
Plasma local anesthetic concentration-time profiles. Arterial plasma levels of bupivacaine (dark blue circles, 4 mg/kg) and ropivacaine (green circles, 5 mg/kg) were quantified at baseline, 1 min after bolus administration, and at 5, 15, and 25 min during intravenous lipid emulsion (ILE) therapy. The arrow indicates the initiation of ILE rescue (3 min post-bolus). Despite the initial difference in peak concentrations due to the equipotent doses administered, integrated Area Under the Curve (AUC 0–25 min) analysis showed no statistically significant differences in total anesthetic exposure between the two groups (p = 0.87). Data are expressed as medians with interquartile ranges (25th–75th percentiles).

Baseline characteristics, including body weight and total procedure time, were similar between groups (Table 1). Subjects maintained physiological stability throughout the protocol, with no significant differences observed in arterial pH, PaCO2, base excess, or electrolyte levels (Na+, K+, and Ca 2+) between the two cohorts. While 100% O2 ventilation induced hyperoxia in both groups, higher PaO2 values were recorded in the ropivacaine group (*p* < 0.05). However, these values remained within supra-physiological ranges and did not result in metabolic or acid-base imbalances. Plasma concentrations of both local anesthetics declined progressively following the bolus. Integrated pharmacokinetic analysis (AUC 0–25 min) confirmed no significant differences in total drug exposure between groups (*p* = 0.87; Figure 3), ensuring that recovery differences were not due to disparate plasma levels.

**TABLE 1:** Laboratory and physiological evaluation at baseline and post-ILE administration.

Parameters	Bupivacaine (n = 6)	Ropivacaine (n = 6)	P value
Weight (kg)	39 (36–41)	41 (40–42)	0.19
Height (cm)	116 (110–125)	114 (111–115)	0.63
Procedure time (min)	163 (154–186)	180 (173–189)	0.41
pH baseline	7.53 (7.49–7.54)	7.52 (7.50–7.53)	0.40
pH 15 min-ILE	7.49 (7.48–7.53)	7.49 (7.47–7.50)	0.32
pH 30 min-ILE	7.49 (7.44–7.52)	7.45 (7.44–7.48)	0.12
PaO_2_ baseline (mmhg)	461 (424–495)	531 (490–565)	0.03
PaO_2_ 15 min-ILE (mmhg)	431 (372–517)	549 (533–567)	0.01
PaO_2_ 30 min-ILE (mmhg)	479 (341–575)	583 (562–598)	0.05
PaCO_2_ baseline (mmhg)	40 (39–44)	38 (35–40)	0.10
PaCO_2_ 15 min-ILE (mmhg)	40 (37–44)	38 (35–42)	0.46
PaCO_2_ 30 min-ILE (mmhg)	39 (37–40)	40 (38–42)	0.68
HCO_3_^−^ baseline (mmol/L)	33 (30–38)	30 (28–33)	0.19
HCO_3_^−^ 15 min-ILE (mmol/L	34 (28–36)	30 (30–32)	0.15
HCO_3_^−^ 30 min-ILE (mmol/L)	32 (29–34)	33 (29–37)	0.63
BE baseline (mmol/L)	10 (7–16)	7 (5–11)	0.26
BE 15 min-ILE (mmol/L)	10 (5–11)	7 (6–8)	0.10
BE 30 min-ILE (mmol/L)	8 (3–10)	5 (4–7)	0.15
SaO_2_ baseline (%)	100 (100–100)	100 (100–100)	1
SaO_2_ 15 min-ILE (%)	100 (100–100)	100 (100–100)	1
SaO_2_ 30 min-ILE (%)	100 (100–100)	100 (100–100)	1
Hemoglobin baseline (gr/dL^-1^)	8.1 (7.8–9)	8.4 (7.9–8.6)	0.74
Hemoglobin 15 min-ILE (gr/dL^-1^)	7.9 (7.2–8.4)	8.4 (7.9–8.9)	0.14
Hemoglobin 30 min-ILE (gr/dL^-1^)	8.1 (7.25–8.25)	8.1 (7.47–8.9)	0.67
Na^+^ baseline (mmol/L)	139 (136–139)	136 (131–140)	0.33
Na^+^ 15 min-ILE (mmol/L)	138 (136–139)	135 (131–137)	0.14
Na^+^ 30 min-ILE (mmol/L)	136 (132–138)	134 (133–137)	0.68
K^+^ baseline (mmol/L)	3.7 (3.45–4.1	3.5 (3.32–3.67)	0.22
K^+^ 15 min-ILE (mmol/L)	3.8 (3.4–4)	3.6 (3.3–4.1)	0.62
K^+^ 30 min-ILE (mmol/L)	4 (3.6–4.2)	3.8 (3.4–3.9)	0.25
Ca^2+^ baseline (mmol/L)	1.26 (1.21–1.32)	1.26 (1.18–1.31)	0.74
Ca^2+^ 15 min-ILE (mmol/L)	1.26 (1.23–1.31)	1.29 (1.20–1.34)	0.81
Ca^2+^ 30 min-ILE (mmol/L)	1.24 (1.18–1.27)	1.27 (1.21–1.29)	0.37

Data are expressed as median (interquartile range). ILE: intravenous lipid emulsion.

PaO_2_ = arterial oxygen partial pressure; PaCO_2_ = arterial carbon dioxide partial pressure; SaO_2_ = arterial oxygen saturation; BE , base excess.

K^+^ = potassium; Na^+^ = sodium; Ca^2+^ = calcium. 15 and 30 min-ILE: 15 and 30 min after the initiation of lipid emulsion therapy.

### Primary outcome: electrophysiological recovery (3–25 min)

**FIGURE 4:**
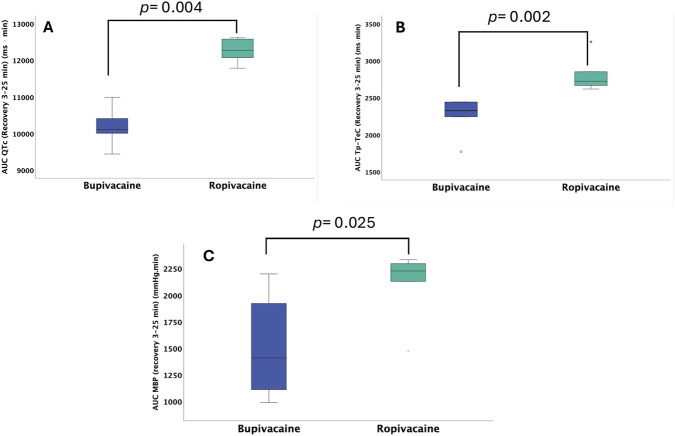
Differential recovery of electrical and mechanical functions following lipid rescue. Integrated recovery analysis using baseline-corrected Area Under the Curve (AUC) during the recovery phase (3–25 min post-intoxication) **(A)** Corrected QT interval (QTc) AUC and **(B)** corrected T-peak to T-end interval (Tp-TeC) AUC. Bupivacaine (blue boxes) exhibited a significantly faster and more effective normalization of ventricular repolarization compared to ropivacaine (green boxes) **(C)** Mean blood pressure (MBP) AUC. Ropivacaine showed a significantly more robust hemodynamic recovery compared to bupivacaine. Data are presented as boxplots (median, interquartile range, and 5th–95th percentiles). Outliers are indicated by individual points. p-values from Mann-Whitney U tests are shown above the brackets. Units: milliseconds minute (msmin) for electrical parameters and millimeters of mercury minute (mmHgmin) for hemodynamics**.**

Following the initiation of lipid resuscitation therapy, a distinct pharmacological divergence was observed. The primary outcome, the recovery phase AUC for Tp-TeC, was significantly lower in the bupivacaine group compared to the ropivacaine group [2,326 (2,125–2,444) msmin vs. 2,723 (2,654–2,954) msmin; *p* = 0.002; Figure 4B]. Similarly, QTc recovery was significantly more efficient in the bupivacaine group, as evidenced by a lower recovery AUC [10,111 (9,863–10,553) msmin vs. 12,272 (12,000–12,589) msmin; *p* = 0.004; Figure 4A]. By the end of the 25-min period, Tp-TeC had nearly normalized in bupivacaine-intoxicated animals [92 (79–98) ms], while it remained elevated in the ropivacaine group [99 (89–104) ms] (Table 2).

**TABLE 2:** Area Under the Curve (AUC) analysis of ventricular repolarization and depolarization markers.

Parameter (unit)	Period	Bupivacaine (n = 6)<	Ropivacaine (n = 6)	P-value
SCL (ms.min)	Toxicity (0–3 min)	1,895 (1,649–2,126)	2,045 (1,889–2,240)	0.26
	Recovery (3–25 min)	14,288 (12,843–16,346)	14,212 (12481–15,654)	0.63
QRS (ms·min)	Toxicity (0–3 min)	296 (256–331)	315 (302–374)	0.26
	Recovery (3–25 min)	2,144 (2,004–2,866)	2,700 (2,535–2,835)	0.07
QT (ms.min)	Toxicity (0–3 min)	1,111 (1,020–1,249)	1,309 (1,230–1,363)	0.055
	Recovery (3–25 min)	8,218 (7,699–9,469)	9,969 (9,423–10,531)	0.055
QTc (ms·min)	Toxicity (0–3 min)	1,401 (1,359–1,537)	1,562 (1,518–1,610)	0.055
	Recovery (3–25 min)	10,111 **(**9,863–10553**)**	12,272 **(**12,000–12,589**)**	0.004*
QTD (ms·min)	Toxicity (0–3 min)	153 (95–216)	228 (174–268)	0.12
	Recovery (3–25 min)	1,227 (993–1,408)	1,325 (896–1,641)	0.52
Tp-Te (ms·min)	Toxicity (0–3 min)	220 (206–286)	325 (298–342)	0.055
	Recovery (3–25 min)	1,856 (1,716–2,067)	2,270 (2,199–2,364)	0.055
Tp-TeC (ms·min)	Toxicity (0–3 min)	318 (274–375)	401 (350–418)	0.109
	Recovery (3–25 min)	2,326 **(**2,125–2,444)	2,723 **(**2,654–2,954**)**	0.002*
Tp-TeD (ms·min)	Toxicity (0–3 min)	150 (110–212)	182 (137–224)	0.63
	Recovery (3–25 min)	1,204 (881–1,285)	1,080 (829–1,346)	1

Data are expressed as median (interquartile range, 25th–75th percentile); n=6 animals per group. Values for AUC are expressed in millisecondsminute (msmin). The Toxicity phase represents the interval from local anesthetic administration to the start of intravenous lipid emulsion (ILE) treatment (0–3 min). The Recovery phase represents the interval from the start of ILE therapy until the end of the 25-min follow-up (3–25 min). Intergroup comparisons (Bupivacaine vs. Ropivacaine) were performed using the Mann-Whitney U test.

* Indicates a statistically significant difference (p < 0.05).

### Secondary outcome: hemodynamic recovery (3–25 min)

**FIGURE 5:**
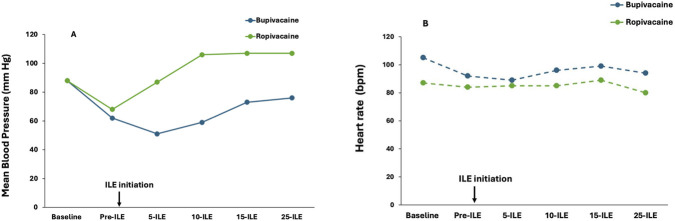
Visual traces of physiological parameters during lipid resuscitation therapy **(A)** Mean blood pressure (MBP) and **(B)** Heart rate. Data points represent medians. The initiation of intravenous lipid emulsion (ILE) occurred immediately after the “Pre-ILE” measurement (3 min post-bolus). Note the distinct supra-recovery of MBP in the ropivacaine group (green line), where pressures exceeded baseline values, contrasting with the incomplete recovery in the bupivacaine group (blue line). Heart rate (bpm) followed a similar trend in both groups, supporting a direct cardiotonic effect on contractility rather than chronotropy.

Hemodynamic recovery was significantly more robust in the ropivacaine group (Table 3; Figure 5C). In bupivacaine-intoxicated animals, MBP reached 76 (58–93) mmHg at 25 min, remaining below baseline levels. In contrast, the ropivacaine group exhibited a supra-recovery profile (Figure 5), with MBP reaching 107 (93–109) mmHg and exceeding baseline values by the end of the study. Accordingly, the recovery AUC for MBP was significantly higher in the ropivacaine group compared to the bupivacaine group [2,106 (1,980–2,290) vs. 1,408 (1,079–1,991) mmHgmin; *p* = 0.025] (Figure 4C). Heart rate recovery followed a similar pattern in both groups (Figure 5), with no significant differences in recovery AUC (*p* = 0.63).

**TABLE 3:** Area Under the Curve (AUC) analysis of hemodynamic parameters.

Parameter (mmHg·min)	Period	Bupivacaine (n = 6)	Ropivacaine (n = 6)	P-value
SBP	Toxicity (0–3 min)	272 (243–293)	295 (271–313)	0.10
	Recovery (3–25 min)	1827 (1,503–2,413)	2,654 (2,453–2,813)	0.010*
DBP	Toxicity (0–3 min)	169 (146–196)	176 (150–212)	0.63
	Recovery (3–25 min)	1,180 (853–1,675)	1,869 (1,457–1,951)	0,10
MBP	Toxicity (0–3 min)	213 (182–238)	226 (204–250)	0.423
	Recovery (3–25 min)	1,408 (1,079–1,991)	2,226 (1,965–2,304)	0.025*

Data are expressed as median (interquartile range, 25th–75th percentile); n=6 animals per group. Values for AUC are expressed in millimeters of mercury minute (mmHgmin). The Toxicity phase represents the interval from local anesthetic administration to the start of intravenous lipid emulsion (ILE) treatment (0–3 min). The Recovery phase represents the interval from the start of ILE therapy until the end of the 25-min follow-up (3–25 min). Intergroup comparisons (Bupivacaine vs. Ropivacaine) were performed using the Mann-Whitney U test. Abbreviations: SBP, systolic blood pressure; DBP, diastolic blood pressure; MBP, mean blood pressure.

* indicates a statistically significant difference ( p<0.05 ) between the bupivacaine and ropivacaine groups during the recovery phase.

### Toxicity induction phase (0–3 min)

**FIGURE 6:**
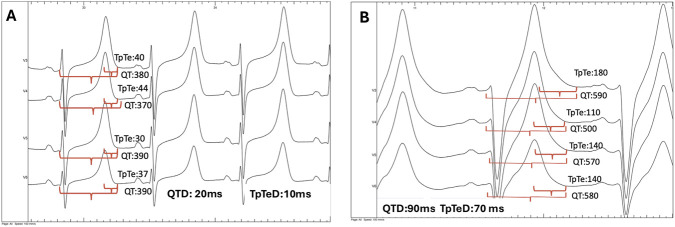
Representative 12-lead ECG traces during the peak of ropivacaine toxicity (3 min post-bolus) **(A)** Baseline and **(B)** peak intoxication traces from a single subject. The labels and red calipers, based on the methodology described in Figure 2, illustrate the measurement of QT and Tp-Te intervals. Note the dramatic widening of the T-wave and the marked increase in transmural dispersion of repolarization (Tp-TeD) and QT dispersion (QTd) during toxicity. All values are expressed in milliseconds (ms).

The administration of both local anesthetics induced immediate electrophysiological and hemodynamic impairment. At the peak of intoxication (3 min post-bolus), ropivacaine induced a broader spectrum of disturbances, significantly altering all repolarization parameters (QTc, Tp-TeC, and dispersions; *p* < 0.05). Bupivacaine significantly affected QRS duration and transmural repolarization markers (*p* < 0.05). Representative electrocardiographic alterations during peak toxicity are illustrated in Figure 6. Despite these snapshots, no statistically significant differences were found between groups in the total toxic burden (AUC 0−3min) for most parameters (*p* > 0.05), ensuring a comparable baseline of intoxication before the start of lipid resuscitation therapy.

Hemodynamically, both groups exhibited significant MBP declines from baseline to peak toxicity (bupivacaine: 88 [70–95] to 62 [46–71] mmHg; ropivacaine: 88 [80–90] to 68 [49–78] mmHg; both *p* < 0.05).

## Discussion

The primary finding of this study is a distinct pharmacological divergence in the efficacy of lipid resuscitation therapy: while ILE accelerated the normalization of ventricular repolarization disturbances more effectively in bupivacaine-intoxicated subjects, ropivacaine-intoxicated subjects exhibited a more robust and rapid hemodynamic recovery. The use of a divided AUC analysis confirmed that although both anesthetics reached comparable levels of electrophysiological impairment during the initial toxicity phase (0–3 min), their response to rescue followed fundamentally different kinetic profiles during the recovery phase (3–25 min).

### The role of lipophilicity and the “lipid shuttle”

The significantly lower recovery AUC for QTc (p = 0.004) and Tp-TeC (p = 0.002) in the bupivacaine group suggests a more efficient reversal of the electrical toxic burden. This differential response is driven by the agents’ distinct physicochemical properties. While Log P is a traditional marker of lipophilicity, the distribution coefficient at physiological pH (Log D at pH 7.4) is more representative of drug behavior during systemic toxicity. Bupivacaine’s higher Log D (≈2.63) compared to ropivacaine (≈2.12) facilitates a more favorable partitioning of the drug into lipid droplets (French et al., 2011; Graf et al., 2002).

Current evidence supports a dynamic “lipid shuttle” model rather than a static “sink” (Fettiplace and Weinberg, 2018). In this model, ILE partitions the lipophilic drug from high-blood-flow tissues and facilitates its redistribution to storage and detoxification organs. The higher affinity of bupivacaine for the lipid phase likely accelerates its dissociation from cardiac ion channels, specifically the hERG potassium channel (responsible for the I_*Kr*_ current) and voltage-gated sodium channels (González et al., 2002; Mio et al., 2004). Conversely, the lower lipophilicity of ropivacaine limits this partitioning effect, resulting in more persistent electrical impairment.

However, these findings must be framed within the contemporary multi-modal understanding of ILE (Lee and Sohn, 2023). Our results suggest a mechanistically-driven divergence: while the shuttle effect (governed by Log D) is the primary determinant for restoring electrical stability, the overall rescue also recruits direct cardiotonic and metabolic pathways (Fettiplace and Weinberg, 2018). This synergy explains why a more lipophilic agent (bupivacaine) shows superior electrical rescue, while a less cardiotoxic agent (ropivacaine) benefits more from the metabolic and inotropic support provided by the emulsion.

### Tp-TeC as a marker of arrhythmogenic risk

Increased myocardial repolarization heterogeneity is a well-established predictor of malignant arrhythmias (Antzelevitch et al., 1999) The Tp-Te interval measures transmural dispersion of repolarization (TDR), reflecting the distinct repolarization time courses between epicardial cells (aligning with the T-wave peak) and midmyocardial M cells (corresponding to the T-wave end). Our data show that ropivacaine, at a clinically equipotent toxic dose, significantly prolongs the Tp-TeC interval, creating a potentially dangerous “arrhythmogenic window.”

While bupivacaine and ropivacaine inhibit hERG channels with similar potency (IC_50_ ≈ 20 μM) (González et al., 2002), the more extensive impairment observed in the ropivacaine group likely stems from the higher total drug load (5 mg/kg vs. 4 mg/kg) required to reach clinical equipotency (Knudsen et al., 1997). However, bupivacaine-induced electrical instability was more rapidly mitigated by lipid resuscitation therapy. The higher lipophilicity of bupivacaine (Log D 2.63 vs. 2.12 for ropivacaine) facilitates more favorable partitioning into the lipid phase. This enhances the lipid shuttle effect, effectively reducing the duration of the period during which the heart is most vulnerable to reentrant arrhythmias.

### The hemodynamic divergence: mechanical vs. electrical recovery

The divergence between the superior electrical recovery of bupivacaine and the robust hemodynamic rescue of ropivacaine (p = 0.025) highlights the multi-modal nature of ILE. Local anesthetics inhibit carnitine-acylcarnitine translocase (CACT), a mitochondrial enzyme critical for fatty acid oxidation (Weinberg et al., 2000; Ok et al., 2018). However, bupivacaine is a more potent inhibitor of this “metabolic gatekeeper,” inducing a deeper state of energy failure. Consequently, the bupivacaine-intoxicated heart may be less capable of utilizing the high-energy fuel provided by the lipid emulsion.

Conversely, ropivacaine’s lower intrinsic mechanical cardiotoxicity and less potent CACT inhibition leave the myocardium more resilient. This allows the heart to effectively harness the direct inotropic and metabolic benefits of ILE, specifically the attenuation of mitochondrial dysfunction and improved calcium signaling (Ok et al., 2018). This synergy explains the supra-recovery profile of MBP observed in the ropivacaine group, where values exceeded baseline (Fettiplace and Weinberg, 2018), even while electrical repolarization remained partially impaired.

Finally, although the porcine model is susceptible to CARPA—an inherent flaw that may confound hemodynamic observations—its anatomical and functional resemblance to the human heart remains valuable for electrophysiological assessment. The stability of the sinus cycle length (p = 0.63) and our previous validation data (Zaballos et al., 2019) suggest that the observed recovery kinetics primarily reflect the pharmacological interaction between ILE and the local anesthetics.

### Limitations

This study has some limitations. First, it was conducted in a healthy porcine model under controlled conditions, which may not fully replicate the complexity of LAST in comorbid human patients. Second, the 25-min observation period, although sufficient to capture the recovery kinetics of ventricular repolarization in this model (Zaballos et al., 2019), may be too short to assess late-phase electrophysiological stability or potential toxicity recurrence.

Third, the use of a porcine model is a significant limitation due to its high susceptibility to CARPA following lipid infusion, a known flaw that can induce transient hemodynamic instability and potentially confound cardiovascular observations (Bedocs et al., 2014). Despite this inherent weakness, the pig remains a common standard for studying transmural dispersion of repolarization due to its anatomical and ion-channel similarities to the human heart. Nevertheless, our findings should be extrapolated to the clinical setting with caution.

## Conclusions

In conclusion, our study demonstrates a distinct pharmacological divergence in the efficacy of lipid resuscitation therapy. While ILE more rapidly mitigates the electrophysiological disturbances induced by bupivacaine, likely due to its higher lipophilicity (Log D) facilitating drug partitioning, ropivacaine-intoxicated subjects exhibit a more robust hemodynamic recovery. This superior mechanical rescue reinforces the multi-modal mechanism of ILE. Specifically, when mitochondrial carnitine transport is less severely inhibited, the heart can better utilize the direct cardiotonic and metabolic support provided by the lipid emulsion. These findings highlight that electrical and mechanical rescue domains are governed by different pharmacological rules.

## Data Availability

The raw data supporting the conclusions of this article will be made available by the authors, without undue reservation.
